# Plasma/Serum Electrolyte and Metabolite Testing on Blood Gas Analyzer ABL837, a New Application †

**DOI:** 10.3390/diagnostics15151923

**Published:** 2025-07-31

**Authors:** Vera Y. Chen, Rachel Fullarton, Yu Chen

**Affiliations:** 1 Department of Laboratory Medicine, Dr. Everett Chalmers Regional Hospital, Horizon Health Network, Fredericton, NB E3B 5N5, Canada; vera.chen@unb.ca (V.Y.C.); rachel.fullarton@horizonnb.ca (R.F.); 2 Faculty of Science, University of New Brunswick, Fredericton, NB E3B 5A3, Canada; 3 Department of Pathology, Dalhousie University, Halifax, NS B3H 4R2, Canada; 4 Discipline of Laboratory Medicine, Memorial University of Newfoundland, St. John’s, NL A1C 5S7, Canada

**Keywords:** blood gas, electrolyte, metabolite, plasma, serum, comparison study

## Abstract

**Background**: Core laboratory chemistry analyzers typically use plasma and serum samples, while blood gas instruments use whole blood for electrolyte and metabolite tests. Due to high costs to back up the core lab chemistry analyzers, especially in the remote small community hospitals, we have verified the interchangeability of serum/plasma electrolytes and metabolites on blood gas instruments (GEM4000 and Radiometer ABL90) vs. chemistry analyzers. In this study, we sought to extend the investigation to another blood gas device—Radiometer ABL837. **Methods**: One plasma separator tube and one serum separator tube were drawn from 20 apparently healthy individuals and outpatients and 20 intensive care unit patients. All the samples were run on Roche Cobas8000, and then were run on three Radiometer ABL837 analyzers for sodium (Na^+^), potassium (K^+^), chloride (Cl^−^), glucose, lactate (plasma only), and creatinine parameters. Paired measurements between the ABL837 and Cobas8000 were compared, and their difference were assessed for statistical and clinical significance. **Results**: ABL837 demonstrated statistical significance (*p* < 0.05) vs. Cobas8000 on all the plasma and serum parameters. However, no parameter differences were found when comparing the plasma/serum results on ABL837 to those on Cobas8000, indicating that none were clinically significant. ABL837 also demonstrated good–excellent correlations with Cobas8000 on all the parameters. **Conclusions**: When comparing metabolite and electrolyte values with plasma and serum sample types, the ABL837 blood gas instruments and Cobas 8000 chemistry analyzer are interchangeable. These data proves that ABL837 can be used as a backup for a chemistry analyzer in measuring plasma and serum electrolyte and metabolite concentrations.

## 1. Introduction

Blood gas (BG) analysis is a commonly utilized laboratory service and is available in most clinical laboratories, satellite laboratories, and point-of-care settings. The fast turnaround time and small sample volume for traditional acid–base and oxygenation testing make BG essential in the workup of patients in severe conditions. With the add-on of electrolyte and metabolite measurement functions, blood gas analyzers (BGAs) play an even more important role in laboratory medicine and healthcare. These new features have gone through phases of untrust, caution, and acceptance by clinicians and laboratorians [1,2,3]. With the advance of technology, the role of BGAs may still be underestimated as new electrolytes and metabolites such as iMg^2+^, creatinine, urea, etc., are being added to their menus.

In addition to the default whole blood sample type, serum and plasma have become sample types of interest on BGAs. Analytically, the value of testing undiluted serum/plasma on BGAs that use direct ion-selective electrodes (ISEs) helps to identify pseudohyponatremia (due to significant elevation of proteins or lipids) on a core laboratory chemistry analyzer that uses indirect ISEs on diluted serum/plasma. However, many medical laboratories have not learned that plasma or serum can be run on BGAs [4]. Furthermore, as hemolysis cannot be detected in whole blood easily, running serum or plasma samples assists with proper interpretation of hyperkalemic or hypokalemic results [4,5]. More importantly, expanding the sample type to serum and plasma on BGAs may provide a rapid backup solution for core laboratory chemistry analyzers for electrolyte (sodium, potassium, and chloride) and metabolite (glucose and lactate) testing without another sample draw. A core lab chemistry analyzer is fairly expensive for many community hospitals, especially for resource-limited facilities and rural areas. Without a backup, those hospitals/labs may have to refer their plasma/serum samples to major centers when their chemistry analyzer breaks down or under routine maintenance, which can be a long duration with delayed turnaround times and lead to potential patient safety risks. As BGAs are common in laboratories as inexpensive analyzers for critical testing, there is a significant socioeconomic value as well as a clinical benefit to validate a new application to run plasma or serum on BGAs for electrolyte and metabolite measurements. Due to higher per-reportable test cost, BGAs are not intended to replace core lab chemistry analyzers for high-volume routine tests for these electrolytes and metabolites.

Our previous study found that the off-label use of plasma/serum on the blood gas analyzer GEM4000 (Instrumentation Laboratory) produced more compatible results for electrolytes (Na^+^, K^+^, and Cl^−^) and metabolites (glucose and lactate) with the Roche Modular P main chemistry analyzer than with BGAs’ default whole blood samples [6]. We also reported that Radiometer ABL90 blood gas analyzers and Roche c501 chemistry analyzer were interchangeable when comparing electrolytes (Na^+^, K^+^, and Cl^−^) and metabolites (glucose, lactate, and plasma tCO_2_) values on plasma and serum sample types [7].

In the current study, we investigated the interchangeability of another BG analyzer–Radiometer ABL837 and a core lab chemistry analyzer—Roche Cobas8000—by comparing the electrolyte and metabolite testing conducted on plasma/serum. Similarly to Roche Cobas8000’s enzymatic glucose and lactate methods, ABL837 measurements take place using glucose oxidase and lactate oxidase methods respectively, followed by an amperometric measuring principle. For accurate lactate level measurements, while both serum and plasma lactate levels may be used in clinical settings, plasma is generally preferred over serum. The blood clotting process can raise lactate levels; therefore, plasma lactate levels are more reflective of the body’s true lactate concentration. As a routine practice in our laboratory, we only compared plasma for lactate in the current study.

In addition to Na^+^, K^+^, Cl^−^, glucose, and lactate, for the first time, we also examined creatinine as it was recently introduced on ABL837. Creatinine is a new feature of the ABL800 FLEX series with a few estimated glomerular filtration rate (eGFR) options for selection. Slightly different from Roche Cobas8000’s creatininase/creatinase/sacrosine oxidase/peroxidase method, ABL837 uses creatinine amidohydrolase/creatine amidohydrolase/sacrosine oxidase method with amperometry for creatinine measurement. Plasma and serum samples were tested on 3 Radiometer ABL837 analyzers, and the results were compared with those that were obtained by a Roche Cobas8000 analyzer to determine whether results from these analyzers were interchangeable.

## 2. Materials and Methods

Blood samples (in plasma separator tubes—PST, and serum separator tubes—SST, all obtained from Becton Dickinson, Franklin Lakes, NJ, USA) were collected from 20 apparently healthy laboratory staff and adult outpatients and 20 intensive care unit patients from Dr. Everett Chalmers Regional Hospital, Horizon Health Network (Fredericton, NB, Canada). The PST/SST samples were tested within 5 min after centrifugation on a Roche Cobas8000 (Indianapolis, IN, USA) chemistry analyzer then were transferred to 1 mL blank syringes and immediately injected on 3 Radiometer ABL837 (Copenhagen, Denmark) blood gas analyzers for the sodium (Na^+^), potassium (K^+^), chloride (Cl^−^), glucose, lactate (only tested on plasma), and creatinine parameters.

Excel v2302 (Microsoft, Redmond, WA, USA) and IBM SPSS v28.0 (Chicago, IL, USA) were used for statistical analysis. Due to non-Gaussian distribution (checked by the Shapiro–Wilk test and additionally verified by inspecting normality plots), data was expressed as medians with interquartile ranges (IQR) and was analyzed with the non-parametric Wilcoxon signed-pair matched-rank test. The Roche Cobas8000 results were paired with each of the ABL837 data. *p*-value < 0.05 was set as statistically significant. Pearson’s correlation was used to evaluate relationships of the plasma/serum results by the Cobas8000 with those by ABL837. Differences (bias) were also plotted to determine whether the data values from the two analyzers were interchangeable. The clinical acceptance limits (CALs) were determined as 4 mmol/L for Na^+^, 0.5 mmol/L for K^+^, 4 mmol/L for Cl^−^, 0.4 mmol/L for lactate, 10% or 0.33 mol/L glucose (whichever greater), and 15% or 27 µmol/L (whichever greater) for creatinine, respectively, following the Clinical Laboratory Improvement Amendments requirement [8] and the College of American Pathologists Chemistry/Therapeutic Drug Monitoring survey evaluation criteria [9]. Result acceptability was ultimately judged by the clinical significance against CALs.

This project was approved by the Research Ethics Services of Horizon Health Network (Fredericton, New Brunswick, Canada) as part of a continuous laboratory quality improvement project, and all the participants had provided their informed consent.

## 3. Results

### 3.1. Comparison of ABL837 with Cobas8000 on Plasma Electrolyte and Metabolite Measurements

**Figure 1:**
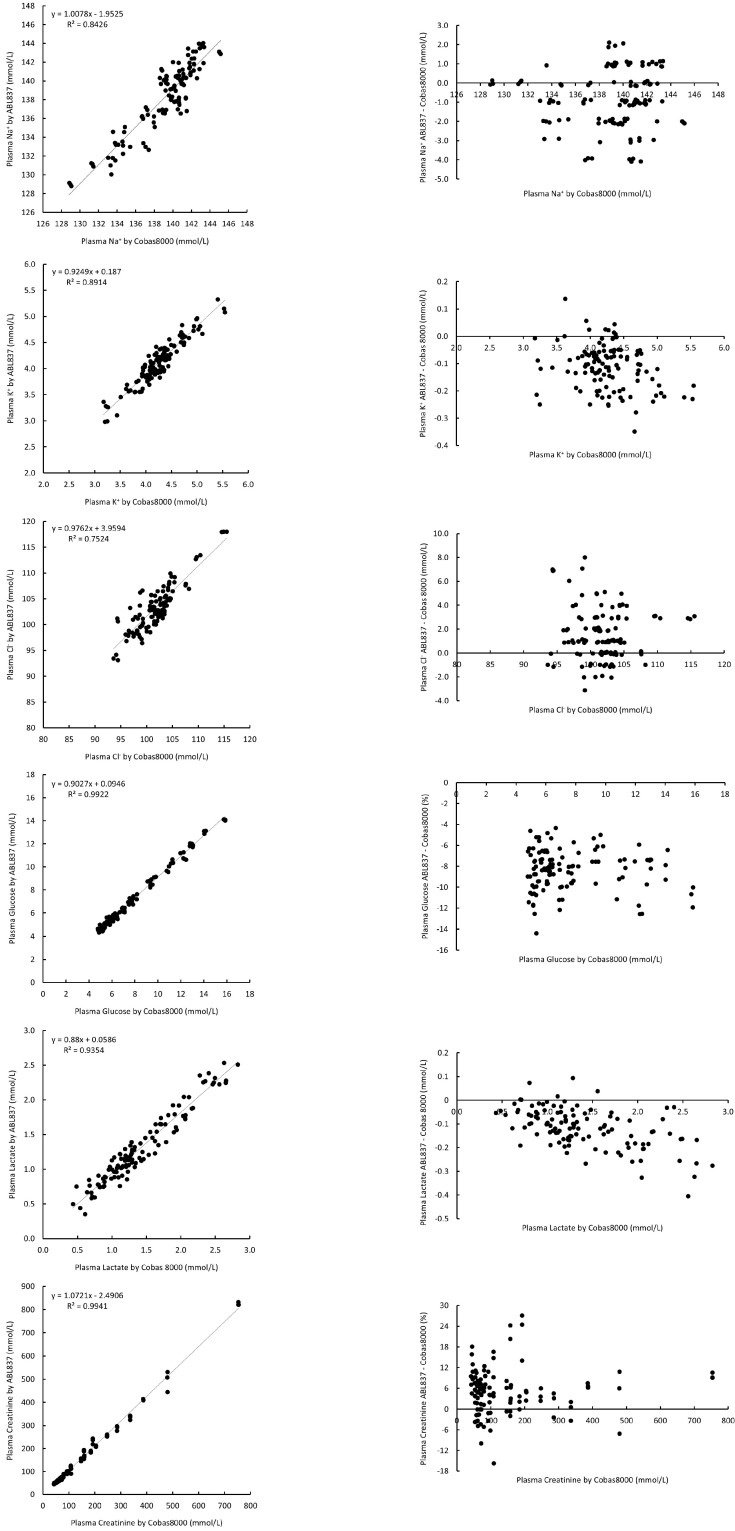
Correlation and parameter difference (bias) for plasma measurements between ABL837 and Cobas8000.

ABL837 has demonstrated excellent correlation with Cobas8000 on plasma Na^+^, K^+^, glucose, lactate, and creatinine (correlation coefficient R values ranged from 0.9179 to 0.997). Plasma Cl^−^ also showed a good correlation with R value of 0.8674 (Table 1, Figure 1). The biases (median difference (95% confidence interval, C.I.) of plasma analytes were Na^+^: −0.89 (−1.175–(−0.61)) mmol/L; K^+^: −0.11 (−0.13–(−0.10)) mmol/L; Cl^−^: 1.58 (1.19–1.98) mmol/L; glucose: −8.3% (−8.7–(−8.0%)); lactate: −0.12 (−0.13–(−0.10) mmol/L; and creatinine: 7.7% (4.8–10.5%), respectively (Table 1). All these biases were statistically significant (*p* < 0.05); however, when further assessed for clinical significance, all the median difference values were within the CALs for all the parameters. All the Na^+^, K^+^, and lactate values were within CALs. There were only 8/117 (6.8%), 19/116 (16.4%), and 4/116 (3.4%) values outside CALs for Cl^−^, glucose, and creatine, respectively (Figure 1).

### 3.2. Comparison of ABL837 with Cobas8000 on Serum Electrolyte and Metabolite Measurements

**Figure 2:**
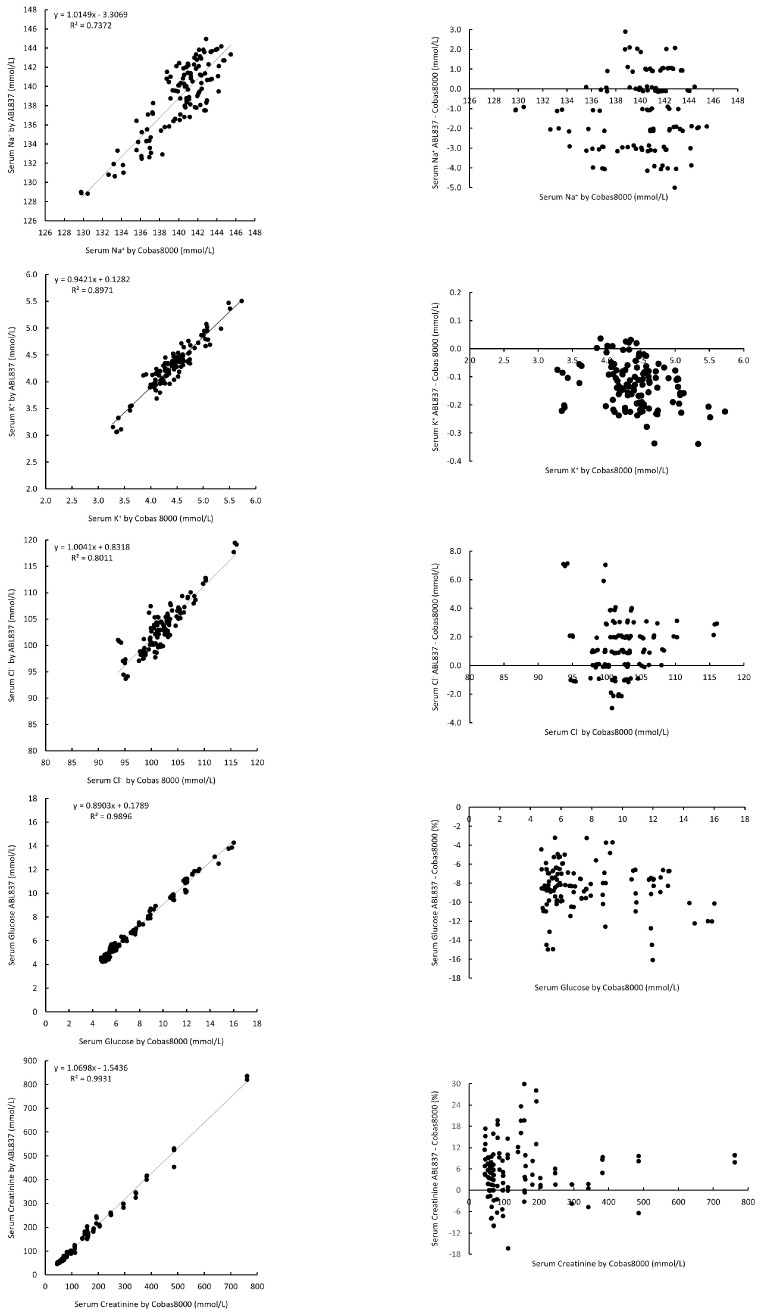
Correlation and parameter difference (bias) for serum measurements between ABL837 and Cobas8000.

Similarly, ABL837 has demonstrated excellent correlation with Cobas8000 on serum K^+^, glucose, and creatinine (correlation coefficient R values were from 0.9472 to 0.9965). Serum Na^+^ and Cl^−^ also showed good correlations with R values of 0.8586 and 0.895, respectively (Table 2, Figure 2). The biases (median difference (95% C.I.) of serum analytes were Na^+^: −1.25 (−1.59–(−0.90)) mmol/L; K^+^: −0.13 (−0.14–(−0.11)) mmol/L; Cl^−^: 1.23 (0.88–1.58) mmol/L; glucose: −8.7% (−9.1–(−8.2%)); and creatinine: 8.4% (5.4–11.3%) (Table 2). All these biases were statistically significant (*p* < 0.05); however, when further assessed for clinical significance, all the median difference values were within the CALs for all the parameters. All the K^+^ values were within CALs. There were only 4/118 (3.4%), 5/117(4.3%), 27/119 (24.3%), and 5/119 (4.2%) values outside CALs for Na^+^, Cl^−^, glucose, and creatine, respectively (Figure 2).

## 4. Discussion

The BG analyzer ABL837 has demonstrated good to excellent correlation and accuracy for plasma and serum Na^+^, K^+^, and Cl^−^, lactate (plasma only), glucose, and creatinine against the core laboratory chemistry analyzer Cobas8000. These findings are generally in line with our previous studies on BG analyzers GEM4000 and ABL90 [6,7].

Comparing with our previous studies [6,7], ABL837 showed improved correlation and accuracy on the plasma and serum Na^+^ measurements, in that correlation coefficient R increased from 0.7–0.8 to around 0.9 in the current study (Table 1 and Table 2), and bias decreased from around 3.0 mmol/L [7] to around 1.0 mmol/L (Table 1 and Table 2). Similarly, ABL837 demonstrated improved performance on the plasma and serum Cl^−^ testing against core laboratory chemistry analyzer in that correlation coefficient R increased from 0.76–0.8 in previous study [7] to 0.87–0.90 (Table 1 and Table 2), and bias decreased from 2.0–2.45 mmol/L previously [7] to 1.2–1.6 mmol/L currently (Table 1 and Table 2). These data may indicate the difference in the Cartridge-based BG analyzers, such as ABL90 and GEM4000, vs. the traditional desktop BGAs, such as ABL837. Although the cartridge-based BGAs have advantages of portability with small sizes and affordable prices, and therefore are widely used in the satellite laboratories and point-of-care testing, there is still room to improve on their electrolyte testing performance.

Another advantage of the current study was the inclusion of critically ill patients (n = 20). This broadened the analytical ranges beyond the reference intervals in this comparison study compared with our previous reports [6,7]. The extensive ranges investigated enhanced the assessment of interchangeability of plasma/serum testing on BGAs vs. chemistry analyzer.

Creatinine is a commonly measured parameter to assess renal function in clinical laboratories. Serum/plasma creatinine and eGFR using serum creatinine (eGFR_cr_) is usually the first line test in laboratory evaluation for chronic kidney diseases, but it is less accurate in conditions that include variable creatinine generation (e.g., muscle wasting diseases, amputees, body builders, morbid obesity, vegan diet); medications that inhibits the proximal renal tubular secretion of creatinine, for example, cimetidine, cobicistat, dolutegravir, fenofibrate, ritonavir, and trimethoprim; and situations with extra-renal elimination of creatinine [10]. eGFR using serum/plasma creatinine and cystatin C (eGFR_cr-cys_) has been found to provide a more accurate estimate of GFR than the equations that include serum/plasma creatinine (eGFR_cr_) or cystatin C (eGFR_cys_) alone [11]. Currently, depending on availability, the National Kidney Foundation and the American Society of Nephrology recommend eGFR_cr_, eGFR_cys_, and eGFR_cr-cys_ using CKD-EPI 2021 equations without race coefficient [10]. Nelson et al. have compared canine serum testing on two BGAs–Prime Plus VET and pHOx Ultra, both from Nova Biomedical Corporation, with a core laboratory chemistry analyzer Beckman Coulter AU680. They found that although electrolyte (Na^+^, K^+^, and Cl^−^) results were acceptable, serum creatinine showed unacceptable biases (42.9% and 35.7%, respectively) on these BGAs, significantly higher than the CLIA (15%) or American Society for Veterinary Clinical Pathology (20%) criteria [12]. Interestingly, Lim et al. concluded that another BGA-ABL90 provided equivalent results for serum creatinine with core laboratory chemistry analyzers Siemens ADVIA1800, Beckman Coulter AU5822, Roche Cobas8000, and Hitachi7600 [13]. Creatinine is now available on ABL837 analyzers. Although whole blood is still the default sample type by the manufacturer, Radiometer does provide validation data of plasma and serum creatinine on ABL837 against Roche Integra, Roche Modular, HPLC reference method, and against whole blood testing on ABL837 and Abbott i-STAT [14]. All the internal data of the manufacturer support the acceptability of plasma and serum creatinine on ABL837. To our knowledge, the current study is the first third-party validation on plasma and serum creatinine on ABL837, demonstrating excellent correlation (R values 0.997 and 0.9965) and accuracy (bias 7.7% and 8.4% with only 4/116 (3.4%) and 5/119 (4.2%) values outside CALs) against Cobas8000.

Plasma and serum glucose testing correlated very well with Cobas8000 (R values 0.9961 and 0.9948, respectively); however, there were negative biases (−8.3% and −8.7%, respectively). Although the average biases and majority values were within the CAL, there were quite a few values out 19/116 (16.4%) for plasma and 27/119 (24.3%) for serum. This reminds clinicians and laboratorians of the limitation, and further research with more subjects may be warranted in this parameter. Although all potassium values on ABL837 were within CAL, we noticed that at high levels (5.0–5.5 mmol/L) and low levels (3.0–3.5 mmol/L), ABL837 demonstrates a negative bias of 0.2–0.3 mmol/L. ABL837 performed better in the normal potassium range. This bias may warrant attention for critically ill patient follow-up and further research.

One interesting finding from the current study was that the plasma tests generally outperform the serum ones on both correlation and accuracy (biases and numbers of values outside CALs) (Table 1 and Table 2). This may further support the use of plasma as a routine sample type for clinical chemistry tests, a trend started about 10–15 years ago among global medical laboratories. As we know, serum is a purer sample type without an anticoagulant as an additive and avoids contaminations with cations (NH4^+^, Li^+^, Na^+^, or K^+^), and prevents interference from ethylene diamine tetraacetic acid (EDTA), heparin, and citrate [15]. However, plasma has the advantage of higher yield (by 15–20% more volume) and reduced turnaround time (by avoiding the coagulation process, which usually takes 30 min). In addition, plasma sample type avoids potential interference of coagulation, such as aspiration needle blockage or measurand changes due to cellular metabolism during the clotting process (glucose, total protein, potassium, etc., which may lead to negative biases and positive estimation, respectively) [15]. Blood from patients on anticoagulants may be challenging to generate serum from. Most importantly, plasma is the more physiological sample type reflecting *in vivo* conditions than serum, which does not exist in living people.

The validation of the non-default use of plasma and serum sample types, especially plasma, provides a new application on BG analyzers and greatly enhances the potenital and the convenience of backup function for urgent breakdown or maintenance situations in that PST or SST tubes may be processed on BG analyzers without the necessity of re-collecting a new whole blood syringe/tube. Each medical laboratory should conduct its own risk assessment based on factors such as patient population, clinical services, geography (distance to other laboratory medicine centers), cost, etc. As this new application may be challenged by local regulations concerning off-label use or laboratory-developed test regulations, a thorough in-house validation and documentation is encouraged.

## 5. Conclusions

In conclusion, the ABL837 blood gas analyzers and Cobas8000 chemistry analyzer are generally interchangeable when comparing metabolite (lactate, glucose, and creatinine) and electrolyte (Na^+^, K^+^, and Cl^−^) values with plasma and serum sample types. Blood gas instruments may serve as a potential backup for core chemistry analyzers for these critical plasma/serum tests.

## Figures and Tables

**Table 1:** Comparison of plasma measurements between ABL837 and Cobas8000.

Analytes	ABL837 Measurement (n)	Slope	Intercept	Correlation Coefficient	ABL837 Median (IQR)	ABL837 Range	Cobas8000 Median (IQR)	Cobas8000 Range	Median Difference (95% C.I.)	*p*-Value
Na^+^ (PST), mmol/L	117	1.0078	−1.9525	0.9179	139 (5.0)	15	140 (4.0)	16	−0.89 (−1.175–(−0.61))	<0.05
K^+^ (PST), mmol/L	117	0.9249	0.187	0.9441	4.1 (0.5)	2.1	4.2 (0.4)	2.1	−0.11 (−0.13–(−0.10))	<0.05
Cl^−^ (PST), mmol/L	117	0.9762	3.9594	0.8674	103 (4.0)	25	102 (4.0)	21	1.58 (1.19–1.98)	<0.05
Glucose (PST), mmol/L	116	0.9027	0.0946	0.9961	5.9 (3.5)	9.8	6.3 (3.7)	11.0	−8.3% (−8.7–(−8.0%))	<0.05
Lactate (PST), mmol/L	114	0.88	0.0586	0.9672	1.15 (0.73)	1.9	1.25 (0.73)	2.1	−0.12 (−0.13–(−0.10))	<0.05
Creatinine (PST), µmol/L	116	1.0721	−2.4906	0.9970	89 (114)	788	80 (99)	711	7.7% (4.8–10.5%)	<0.05

C.I.: confidence interval; IQR: interquartile range; n: number; PST: plasma separator tube.

**Table 2:** Comparison of serum measurements between ABL837 and Cobas8000.

Analytes	ABL837 Measurement (n)	Slope	Intercept	Correlation Coefficient	ABL837 Median (IQR)	ABL837 Range	Cobas8000 Median (IQR)	Cobas8000 Range	Median Difference (95% C.I.)	*p*-Value
Na^+^ (SST), mmol/L	118	1.0149	−3.3069	0.8586	140 (5.0)	16	141 (3.0)	15	−1.25 (−1.59–(−0.90))	<0.05
K^+^ (SST), mmol/L	119	0.9421	0.1282	0.9472	4.3 (0.5)	2.2	4.4 (0.5)	2.3	−0.13 (−0.14–(−0.11))	<0.05
Cl^−^ (SST), mmol/L	117	1.0041	0.8318	0.8950	103 (5.0)	25	102 (3.0)	22	1.23 (0.88–1.58)	<0.05
Glucose (SST), mmol/L	119	0.8903	0.1789	0.9948	5.6 (3.4)	13.2	6.0 (3.8)	14.6	−8.7% (−9.1–(−8.2%))	<0.05
Creatinine (SST), µmol/L	119	1.0698	−1.5436	0.9965	91 (120)	798	81 (101)	717	8.4% (5.4–11.3%)	<0.05

C.I.: confidence interval; IQR: interquartile range; n, number; SST: serum separator tube.

## Data Availability

The data underlying this article are available in the article.
